# Biomechanical analysis of a trans-discal, multi-level stabilization screw (MLSS) at the upper instrumented vertebra (UIV) of long posterior thoracolumbar instrumentations

**DOI:** 10.1007/s43390-024-00862-7

**Published:** 2024-04-05

**Authors:** Andrew P. Collins, Anoli A. Shah, Niloufar Shekouhi, Vijay K. Goel, Alekos A. Theologis

**Affiliations:** 1 Department of Orthopaedics and Sports Medicine, University of Washington, Seattle, WA USA; 2 Engineering Center for Orthopaedic Research Excellence (E-CORE), Departments of Bioengineering and Surgery, Colleges of Engineering and Medicine, University of Toledo, Toledo, OH USA; 3 Department of Orthopaedic Surgery, San Francisco (UCSF), University of California, 500 Parnassus Ave, MUW 3rd Floor, San Francisco, CA USA

**Keywords:** Finite element analysis, Biomechanics, Adjacent segment, Trans-discal screws, Multi-level stabilizing screw

## Abstract

**Purpose:**

To evaluate proximal junctional biomechanics of a MLSS relative to traditional pedicle screw fixation at the proximal extent of T10-pelvis posterior instrumentation constructs (T10-p PSF).

**Methods:**

A previously validated three-dimensional osseoligamentous spinopelvic finite element (FE) model was used to compare proximal junctional range-of-motion (ROM), vertebral body stresses, and discal biomechanics between two groups: (1) T10-p with a T10-11 MLSS (“T10-11 MLSS”) and (2) T10-p with a traditional T10 pedicle screw (“Traditional T10-PS”).

**Results:**

The T10-11 MLSS had a 5% decrease in T9 cortical bone stress compared to Traditional T10-PS. Conversely, the T10 and T11 bone stresses increased by 46% and 98%, respectively, with T10-11 MLSS compared to Traditional T10-PS. Annular stresses and intradiscal pressures (IDP) were similar at T9-T10 between constructs. At the T10-11 disc, T10-11 MLSS decreased annular stresses by 29% and IDP by 48% compared to Traditional T10-PS. Adjacent ROM (T8-9 & T9-10) were similar between T10-11 MLSS and Traditional T10-PS. T10-11 MLSS had 39% greater ROM at T10-11 and 23% less ROM at T11-12 compared to Traditional T10-PS.

**Conclusions:**

In this FE analysis, a T10-11 MLSS at the proximal extent of T10-pelvis posterior instrumentation resulted in increased T10 and T11 cortical bone stresses, decreased discal annular stress and IDP and increased ROM at T10-11, and no change in ROM at the adjacent level. Given the complex and multifactorial nature of proximal junctional kyphosis, these results require additional biomechanical and clinical evaluations to determine the clinical utility of MLSS on the proximal junctions of thoracolumbar posterior instrumented fusions.

## Introduction

Proximal junctional kyphosis (PJK) and fractures (PJF) are common causes of revision operations that jeopardize patient quality of life, improvement in clinical outcomes, cost-effectiveness, and utility of index operations for adult spinal deformity (ASD) [1–3]. Two failure mechanisms constitute PJK/PJF: ligamentous failure and osseous failure [4]. Ligamentous failure often is due to iatrogenic alteration of the posterior supraspinous and interspinous ligaments, spinous process, and paraspinal musculature and can result in catastrophic subluxation at the proximal junction [4–9]. Osseous failure resulting in PJK/PJF presents with a fracture of the upper instrumented vertebrae (UIV) and/or fracture of the supra-adjacent vertebrae (UIV + 1) [4]. Fracture patterns are heterogeneous, ranging from a compression fracture with mild kyphosis, severe UIV collapse, or fracture-dislocation of the UIV + 1 [4]. Translation instability is often clinically significant and commonly results in myelopathy necessitating revision surgery [9].

Minimizing the risk of developing PJK/PJF is complex and multifactorial, including creating a smooth load transfer from the instrumented levels to the un-instrumented at the proximal segments, restoring harmonious sagittal alignment, and minimizing soft tissue disruption at the proximal junction. While multiple techniques have been proposed to achieve this goal, one that is relatively unexplored is a screw placed into the UIV (i.e., T10) through the UIV/UIV-1 disc space (T10-T11) of a T10-pelvis posterior instrumentation construct. This type of thoracic trans-discal screw has been termed a “Multi-Level Stabilization Screw (MLSS)” [10–14]. The purpose of this study is to compare the proximal junctional biomechanics of T10 to pelvis posterior instrumented constructs supplemented with a T10-11 MLSS to traditional pedicle screw fixation at T10.

## Materials and methods

**Fig. 1:**
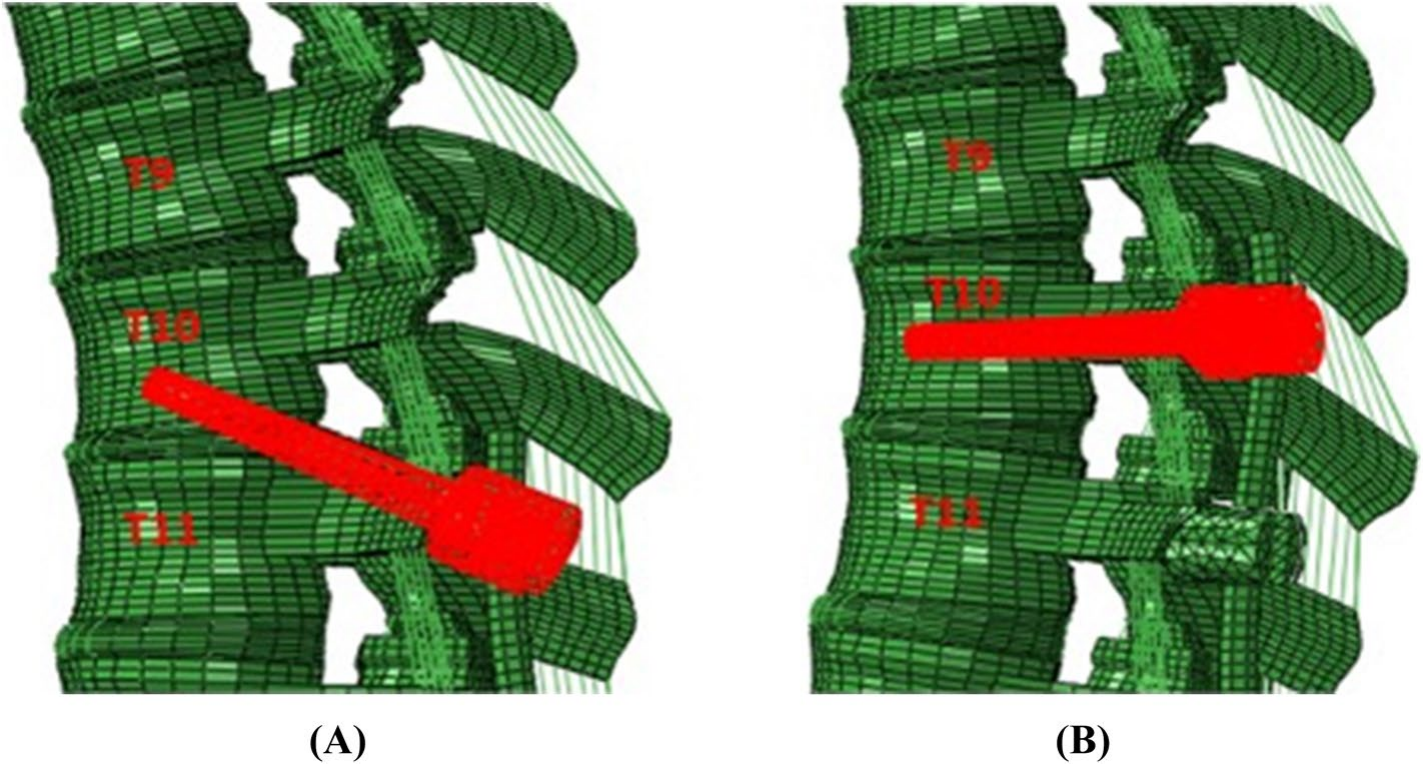
Pictorial examples of (A) T10-11 multi-level stabilizing screws (“MLSS”) and (B) traditional pedicle screw fixation at T10 above T10-pelvis posterior instrumentation constructs

A previously validated three-dimensional osseoligamentous spinopelvic finite element model (T10-pelvis) was used (Fig. 1) [15, 16]. The initial intact model of the ligamentous spine was reconstructed from computed tomography (CT) scans of a human spine using MIMICS (Materialize Inc., Leuven, Belgium) software. The IAFE-MESH (University of Iowa, Iowa) and HyperMesh (Altair Engineering, Michigan, USA) software were used to create hexahedral elements (C3D8) of the vertebrae and tetrahedral elements (C3D4) of the pelvis. The meshed components were assembled in the Abaqus 6.14 (DassaultSystemes, Simulia Inc., Providence, RI, USA) software. The spinal and sacroiliac ligaments were modeled using truss elements. In the vertebral body, a layer of 0.5 mm cortical bone was simulated to surround the cancellous bone.

The material properties for cortical/cancellous bones, annulus, nucleus, ligaments, and joints were selected based on the literature and assigned (Table 1). [17, 24] Isotropic material properties were utilized for bone due to the relatively uniform structure and response to stress in all directions, which is characteristic of bone tissue. The intervertebral discs were composed of annulus fibrosis and nucleus pulposus. To accurately capture the complex behavior of these two components, hyperelastic anisotropic material models were adopted from the literature and employed [17, 24]. The annulus fibrosis was simulated using a solid ground substance (C3D8 elements) that was reinforced with rebar elements (embedded into the ground matrix with ± 30° angles) [17, 24]. The nucleus pulposus was modeled using C3D8 elements with hyper-elastic Mooney-Rivlin formulation. Articular surfaces (sacroiliac joints, spine facets, articular cartilages, and pubic symphysis) were assigned an exponential contact that adjusts the force as the distance between the surfaces decrease [17, 24]. The cartilaginous layer at the sacroiliac joint was simulated using soft contact with force-adjusting exponential behavior [17, 24]. Hypoelastic nonlinear material behavior was defined for each spinal and pelvis ligaments based on their force–deflection curve (Table 1).

**Table 1:** Material Properties Used in Model Development [17, 24]

Components	Constitutive Relation	Element Type	Young’s Modulus (MPa) /Poisson’s Ratio
Vertebral cortical bone	Isotropic, elastic	Hex elements (C3D8)	12,000/0.3
Vertebral cancellous bone	Isotropic, elastic	Hex elements (C3D8)	100/0.2
Pelvic cortical bone	Isotropic, elastic	Hex elements (C3D8)	17,000/0.3
Pelvic cancellous bone	Isotropic, elastic	Hex elements (C3D8)	10/0.2
Annulus (ground)	Non-linear, Neo- Hookean	Hex elements (C3D8)	C10 = 0.348, D1 = 0.3
Annulus (fiber)	Non-linear, Hypo-elastic	Rebar	357 − 550
Nucleus	Hyper-elastic, Mooney Rivlin	Hex elements (C3D8H)	C1 = 0.12, C2 = 0.03, D1 = 0.0005
Apophyseal joints	Nonlinear soft contact	GAPUNI	–
Sacroiliac joints	Nonlinear soft contact		–
Ligaments	Hypo-elastic, tension only	Truss elements (T3D2)	Nonlinear stress − strain curves
Ti6Al4V pedicle screws	Isotropic, elastic	Tetrahedron elements (C3D4)	11,500/0.3
Ti6Al4V rods	Isotropic, elastic	Tetrahedron elements (C3D4)	11,500/0.3

### Models

Two construct models were developed: (1) “Traditional T10-PS”: instrumented with pedicle screws and rods from T10-pelvis and (2) “T10-11 MLSS”: instrumented with pedicle screws and rods from T10-pelvis with the UIV screw placed from T11 into T10 through the T10-T11 disc (Fig. 1). All instrumentation was designed in SolidWorks (Dassault Systems, SolidWorks Corporation, Waltham, MA, USA) software and imported into Abaqus for model development. Each pedicle screw was modeled in a two-part (including a tulip & a shaft) connected with a ball & socket joint. All screws and rods were titanium alloy. Rod diameters were 5.5 mm.

A two-step analysis was performed. In step one, the spine model was pre-loaded with axial compression load to simulate body weight using the following load technique: 300 N to the thoracic spine, 400 N to the lumbar spine, and 400 N to the sacrum [17, 18]. In step two, pure moments of 7.5 Nm were applied to the top endplate of the T10 vertebra in all three anatomical directions. In both steps, the acetabulum surfaces of the pelvis were fixed in all degrees of freedom.

### Outcome measures

For each instrumentation technique, the range of motion (ROM), maximum cortical stresses, intervertebral disc annular stresses, and intervertebral disc pressures (IDP) were evaluated and compared between Traditional T10-PS constructs and T10-11 MLSS constructs.

## Results

### ***Vertebral body stresses*** (Fig. 2)

**Fig. 2:**
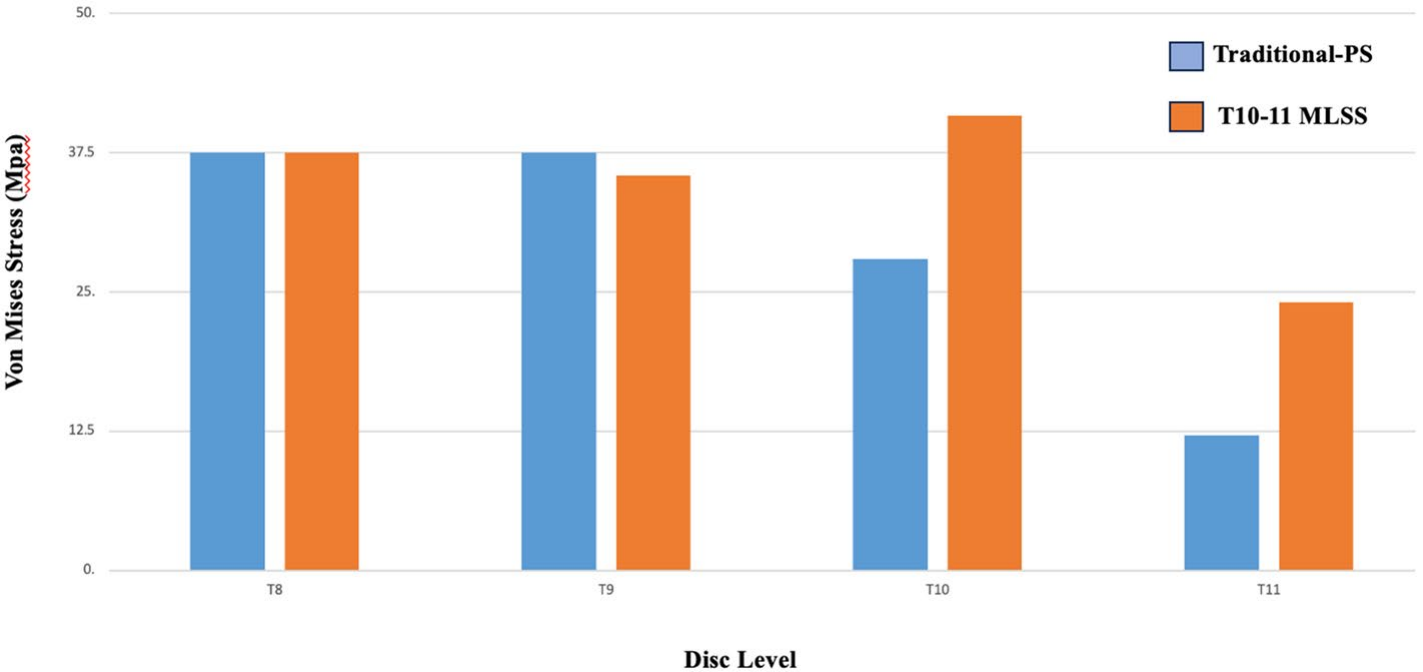
Comparison of the cortical vertebral stress between the T10-11 MLSS and Traditional-PS instrumentation constructs

Stresses at the cortical bone of T9 decreased by 5% in the T10-11 MLSS construct compared to Traditional T10-PS. Conversely, the stresses within the cortical bones of T10 and T11 increased by 46% and 98%, respectively, with the T10-11 MLSS compared to Traditional T10-PS.

### ***Discal biomechanics*** (Figs. 3 and 4)

**Fig. 3:**
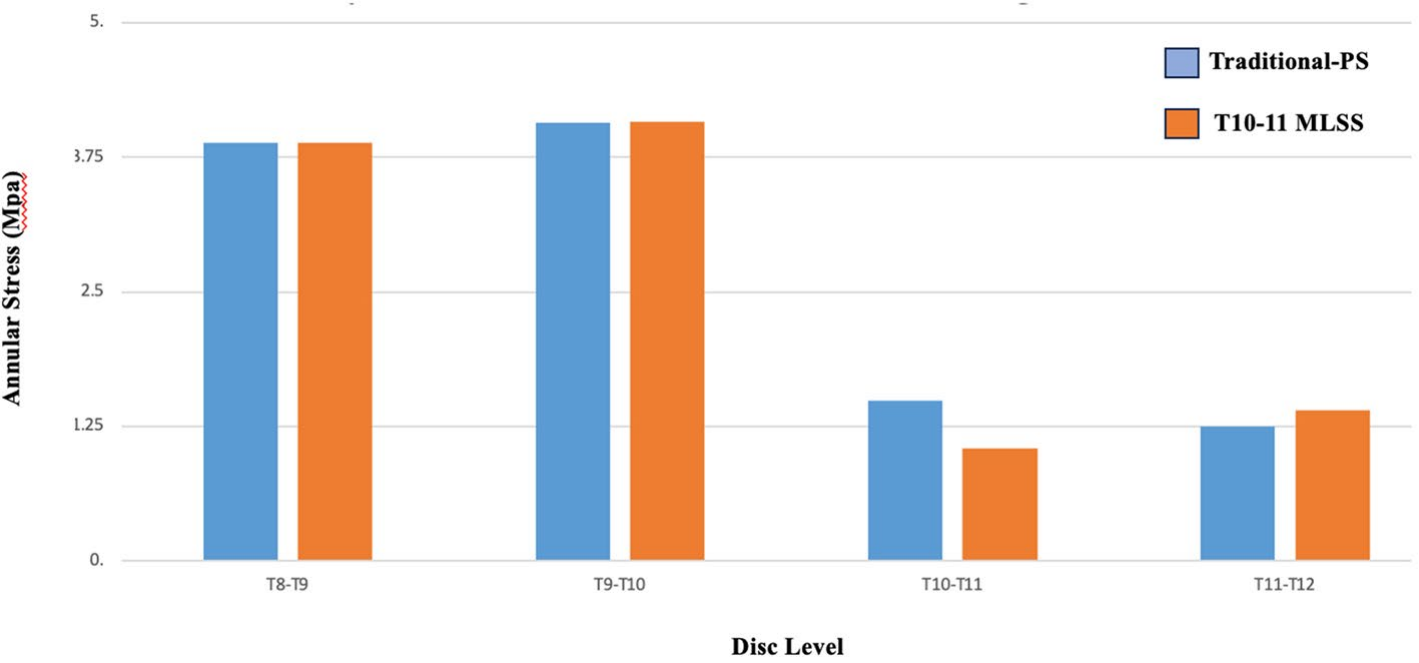
Comparison of the annular stresses between the T10-11 MLSS and Traditional-PS instrumentation constructs

**Fig. 4:**
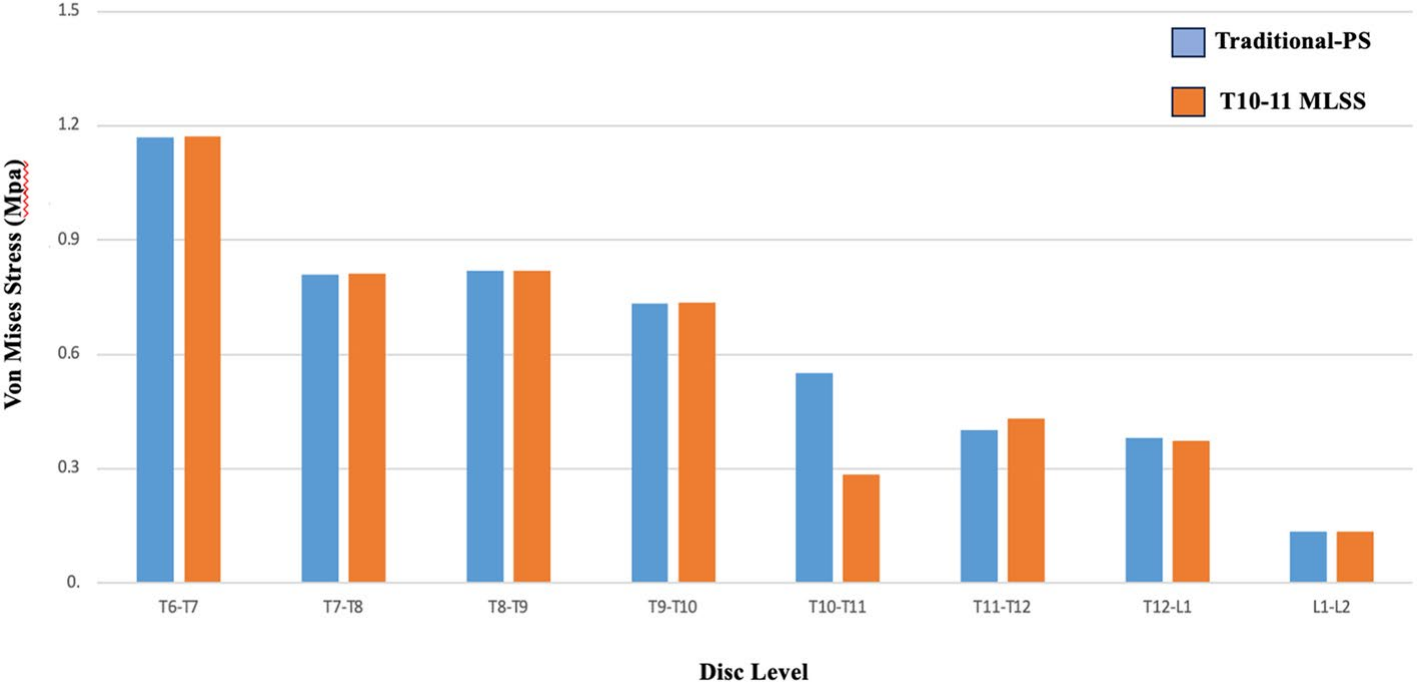
Comparison of the intra-discal pressures (ID) between the T10-11 MLSS and Traditional-PS instrumentation constructs

Annular stresses were similar at the T9-T10 disc between the T10-11 MLSS construct and the Traditional T10-PS construct. Intradiscal pressures were also similar at the T9-T10 disc between T10-11 MLSS and Traditional T10-PS. At the T10-11 disc, T10-11 MLSS was found to decrease annular stresses by 29% and IDP by 48% compared to Traditional T10-PS.

### ***Range of motion*** (Fig. 5)

**Fig. 5:**
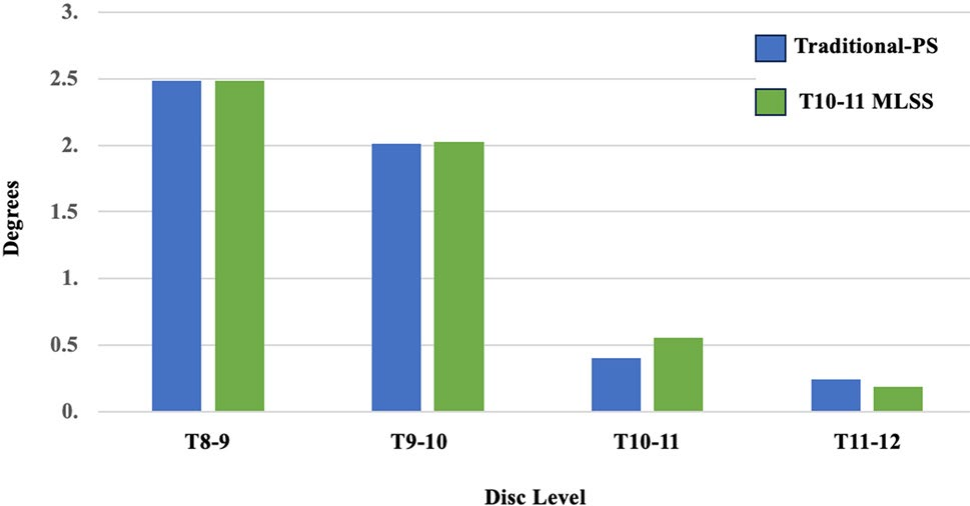
Comparison of range of motions between the T10-11 MLSS and Traditional-PS instrumentation constructs

Range of motion at the T8-9 and T9-10 vertebrae were similar (within 1%) between T10-11 MLSS and Traditional T10-PS. The T10-11 MLSS constructs had 39% greater ROM in the T10-11 segment and 23% less ROM in the T11-12 segment compared to Traditional T10-PS.

## Discussion

In this study, we present for the first time a finite element analysis of proximal junctional biomechanics of a screw placed into the UIV (T10) through the T10-T11 disc at the proximal extent of T10-pelvis posterior instrumentation construct [“Multi-Level Stabilization Screw (MLSS)]” compared to traditional pedicle screw fixation at T10. Our results demonstrated biomechanical differences at the proximal junction between the two constructs in regard to bone stresses, disc parameters, and ROM. Specifically, the T10-T11 MLSS was found to result in slightly lower bone stress at the UIV + 1 (T9) but significantly greater stresses in the UIV (T10; + 46%) and UIV-1 (T11; + 98%) compared to conventional T10 pedicle screw fixation. No differences in adjacent segment discal parameters (T9-10) were found, though annular stresses and IDP decreased notably in the T10-11 disc through which the T10-11 MLSS traversed. Furthermore, ROM at T10-11 increased in the intradiscal screw model by 39% compared to the traditional construct, while ROMs cranially to the UIV (T8-9 and T9-10) were similar between the two models.

The concept of a MLSS was initially described for stabilization of the lumbosacral junction in moderate- to high-grade spondylolisthesis with resultant high fusion rates, low incidence of neurologic complications, decreased operative duration, and more rapid return to activity [19–21]. In a cadaveric biomechanical study at the lumbosacral junction, *Minamide *et al*.* reported 1.6–1.8 times stiffer fixation with transdiscal screws compared to pedicle screw fixation and had no difference in stiffness compared to combined interbody/pedicle screw fixation [22]. In a comparative case–control study assessing outcomes of transdiscal screw versus pedicle screw fixation for high-grade L5-S1 isthmic spondylolisthesis, improved functional and radiographic outcomes at 2-year follow-up were noted in the transdiscal screw cohort [23]. Given this documented safety and efficacy for lumbosacral pathology as well as the perceived advantage that transdiscal pedicle screws increase the stability of a construct as they purchase multiple cortical layers of two vertebrae, the use of MLSS expanded to fixation of thoracic spine pathology.

Documented clinical use of MLSS in the thoracic spine is limited but encouraging. In 2013, *Nottmeier *et al. presented the first case report of 12 patients undergoing cervicothoracic or thoracic fusions in whom MLSS screws were placed using navigation to achieve improved purchase in the setting of osteoporosis, failed pedicle screws, and/or at the proximal extent of the constructs [13]. Use of MLSS has also been reported to be achieved safely via percutaneous techniques in patients with diffuse idiopathic skeletal hyperostosis, and were favored over traditional pedicle screw fixation given a significant reduction in screw loosening and 134% higher insertional torque [10]. The implementation of MLSS in adult deformity surgery at the UIV was first reported in a case series of 20 patients by *Sandquist *et al*.* who found a 0% incidence of PJK and PJF [7]. In turn, it was concluded that MLSS is safe and efficacious in the thoracic spine to reduce the incidence of proximal junctional complications [7]. Our study augments this previously published clinical study through a finite element analysis and provides greater insight into the biomechanics of this specific technique at the proximal junction of the long posterior thoracolumbar junction.

Our findings, in sum, suggest that the T10-T11 MLSS increases the range of motion and lowers intradiscal parameters at the T10-11 segment (relative to traditional pedicle screw fixation), which in turn transitions to an unfused segment. Our ROM findings are consistent with the only other prior biomechanical analysis of thoracic MLSS by *Rodriguez-Martinez *et al. [14]. That there is increased ROM at T10-11, no differences in T9-10 ROM, and considerably increased stresses in the T10 and T11 vertebral bodies in the setting of the T10-11 MLSS may suggest an increased risk of osseous failure/fractures of the UIV. However, that there were no muscle forces simulated may potentially underestimate the biomechanical benefits of the MLSS technique given that the MLSS screw may be less disruptive to the proximal junction’s soft tissues, as its relatively inferior and medial start point allows for preservation of the soft tissue attachments to the lateral aspect of the UIV’s transverse processes. Comparatively, the more cranial and lateral start site of a UIV pedicle screw may be more destabilizing to the soft tissue posterior tension band, which is a known risk factor for developing PJK [5–8].

While the ultimate clinical behavior of the MLSS remains incompletely understood, it is reassuring that the aforementioned cohort presented by *Sandquist *et al*.* did not result in an unacceptably high rate of PJK/PJF [7]. Although *Sandquist *et al*.* did not report any complications associated with the MLSS technique, some potential downsides of the MLSS may include a breach of the UIV’s cranial endplate that may result in accelerated adjacent segment disease, limited fixation of the MLSS in the UIV as a result of a less inclined caudal-cranial trajectory, challenges placing the MLSS screw in the appropriate position by a free-hand technique, increased risk of pseudarthrosis as a result of less rigid fixation across the T10-11 segment, and possibly higher risk of screw pull-out given the cranially directed nature of the screw trajectory [25]. Note these risks are postulations, as they have not been reported in the literature given limited clinical experiences with this technique. Furthermore, additional short- and long-term clinical challenges may be associated with the MLSS technique to which we are not privy. As this rarely used technique may have more potential risks than benefits, additional clinical series would be helpful to ultimately determine if stabilization of the proximal segments of long thoracolumbar fusions with MLSS is safe, effective, and durable.

The results of this study should be considered in the context of its limitations. While we believe the accuracy of this finite element analysis model is acceptable given its use of a well-established, previously validated model, there are several factors that may jeopardize its accuracy in simulating the forces following a long posterior thoracolumbar instrumented fusion. These include simulations performed using uncomplicated geometries of the implants and simplified contacts and constraints as well as no muscle forces. An additional limitation is that proximal junctional biomechanics may be influenced by other factors, including, but not limited to, rod diameter, rod material, sagittal alignment, and bone quality. Moreover, as a sensitivity analysis is the process of evaluating how the output of FE models changes with respect to variations in the input parameters (i.e. material properties, boundary conditions, geometry, mesh size, loads), our model, in turn, may not be generalizable to all clinical scenarios in which different materials are used for different instrumentation strategies (i.e. cobalt chrome rod, titanium rod, pedicle screw with cobalt chrome tulip head and titanium shaft, all titanium pedicle screw, rod sizes 5.5 vs. 6.0 vs. 6.25 mm, etc.). However, it should be noted that while the model has these limitations, the use of comparative analyses (relative to the “Traditional T10-PS” construct) makes our reported relative differences of greater credence than individual absolute values. While we report relative differences between the different screw configurations, we are unable to comment upon the biomechanical and clinical significances of our observed biomechanical differences and relative long-term clinical performance of the different instrumentation configurations evaluated in this study, particularly because the exact margin of error, as well as the margin of important difference, are not known. Despite our model not being able to capture every nuanced difference in instrumentation strategy used clinically, we do feel that there is potential clinical applicability of our data. However, the ultimate determination of the clinical applicability of our findings will require additional clinical investigations. Ideally, the result of this study may be considered a unique addition to the growing literature on a unique fixation strategy (MLSS) at the proximal junction of long posterior instrumented fusions that will stimulate further discussion and inquiry into their clinical utility.

## Conclusions

In this finite element analysis, a screw placed into the UIV (T10) through the T10-T11 disc at the proximal extent of T10-pelvis posterior instrumentation construct resulted in increased cortical bone stresses at T10 and T11, decreased discal parameters (annular stresses and IDP) and increased ROM at T10-11, and no change in ROM at the adjacent level (T9-10). Given the complex and multifactorial nature of PJK/PJF, these results require additional biomechanical evaluations as well as clinical investigations so as to determine clinical utility and in vivo effects of MLSS on the proximal junctions of long thoracolumbar posterior instrumented fusions.

## Data Availability

Data is available on request from the authors.

## References

[1] Riley MS, Bridwell KH, Lenke LG, Dalton J, Kelly MP (2018). Health-related quality of life outcomes in complex adult spinal deformity surgery. J Neurosurg Spine.

[2] Durand WM, Babu JM, Hamilton DK (2022). Adult spinal deformity surgery is associated with increased productivity and decreased absenteeism from work and school. Spine.

[3] Ryu WHA, Cheong M, Platt A (2021). Patient Satisfaction Following Minimally Invasive and Open Surgeries for Adult Spinal Deformity. World Neurosurg.

[4] Yagi M, King AB, Boachie-Adjei O (2012). Incidence, risk factors, and natural course of proximal junctional kyphosis: surgical outcomes review of adult idiopathic scoliosis. Minimum 5 years of follow-up. Spine.

[5] Gupta MC, Wijesekera S, Sossan A (2007). Reliability of radiographic parameters in neuromuscular scoliosis. Spine.

[6] Ha Y, Maruo K, Racine L (2013). Proximal junctional kyphosis and clinical outcomes in adult spinal deformity surgery with fusion from the thoracic spine to the sacrum: a comparison of proximal and distal upper instrumented vertebrae. J Neurosurg Spine.

[7] Sandquist L, Carr D, Tong D, Gonda R, Soo TM (2015). Preventing proximal junctional failure in long segmental instrumented cases of adult degenerative scoliosis using a multilevel stabilization screw technique. Surg Neurol Int.

[8] Vercoulen TFG, Doodkorte RJP, Roth A, de Bie R, Willems PC (2022). Instrumentation techniques to prevent proximal junctional kyphosis and proximal junctional failure in adult spinal deformity correction: a systematic review of clinical studies. Global Spine J.

[9] Fakhre E, Kelly MJ, Mo FF (2022). Proximal junctional kyphosis. Semin Spine Surg.

[10] Takeuchi T, Hosogane N, Yamagishi K, Satomi K, Matsukawa K, Ichimura S (2020). Results of using a novel percutaneous pedicle screw technique for patients with diffuse idiopathic skeletal hyperostosis-the single or double endplates penetrating screw (SEPS/DEPS) technique. Spine Surg Relat Res.

[11] Filis AK, Aghayev K, Schaller B, Luksza J, Vrionis FD (2016). Transdiscal mid- and upper thoracic vertebroplasty: first description of 2 exemplary cases. J Neurosurg Spine.

[12] Mehdizade A, Payer M, Somon T (2004). Percutaneous vertebroplasty through a transdiscal access route after lumbar transpedicular instrumentation. Spine J.

[13] Nottmeier EW, Pirris SM (2013). Placement of thoracic transvertebral pedicle screws using 3D image guidance. J Neurosurg Spine.

[14] Rodriguez-Martinez NG, Savardekar A, Nottmeier EW (2016). Biomechanics of transvertebral screw fixation in the thoracic spine: an in vitro study. J Neurosurg Spine.

[15] Goel VK, Grauer JN, Patel TC (2005). Effects of charité artificial disc on the implanted and adjacent spinal segments mechanics using a hybrid testing protocol. Spine.

[16] Goel VK, Mehta A, Jangra J (2007). Anatomic facet replacement system (AFRS) restoration of lumbar segment mechanics to intact: a finite element study and in vitro cadaver investigation. SAS J.

[17] Seyed Vosoughi A, Joukar A, Kiapour A (2019). Optimal satellite rod constructs to mitigate rod failure following pedicle subtraction osteotomy (PSO): a finite element study. Spine J.

[18] Patwardhan AG, Havey RM, Carandang G (2003). Effect of compressive follower preload on the flexion-extension response of the human lumbar spine. J Orthop Res.

[19] Abdu WA, Wilber RG, Emery SE (1994). Pedicular transvertebral screw fixation of the lumbosacral spine in spondylolisthesis. A new technique for stabilization. Spine.

[20] Logroscino CA, Tamburrelli FC, Scaramuzzo L, Schirò GR, Sessa S, Proietti L (2012). Transdiscal L5–S1 screws for the treatment of adult spondylolisthesis. Eur Spine J.

[21] Rindler RS, Miller BA, Eshraghi SR (2016). Efficacy of transsacral instrumentation for high-grade spondylolisthesis at L5–S1: a systematic review of the literature. World Neurosurg.

[22] Minamide A, Akamaru T, Yoon ST, Tamaki T, Rhee JM, Hutton WC (2003). Transdiscal L5–S1 screws for the fixation of isthmic spondylolisthesis: a biomechanical evaluation. J Spinal Disord Tech.

[23] Collados-Maestre I, Lizaur-Utrilla A, Bas-Hermida T, Pastor-Fernandez E, Gil-Guillen V (2016). Transdiscal screw versus pedicle screw fixation for high-grade L5–S1 isthmic spondylolisthesis in patients younger than 60 years: a case-control study. Eur Spine J.

[24] Lindsey D, Kiapour A, Yerby S, Goel V (2015). Sacroiliac joint fusion minimally affects adjacent lumbar segment motion: a finite element study. Int J Spine Surg.

[25] Harris AB, Kebaish FN, Puvanesarajah V (2021). Caudally directed upper-instrumented vertebra pedicle screws associated with minimized risk of proximal junctional failure in patients with long posterior spinal fusion for adult spinal deformity. Spine J.

